# Losing trust in the world: Humiliation and its consequences

**DOI:** 10.1080/14753634.2013.778485

**Published:** 2013-04-02

**Authors:** Phil Leask

**Affiliations:** Department of German, University College London, London, UK

**Keywords:** humiliation, shame, trauma, abuse, rage, revenge, injustice

## Abstract

The author identifies acts of humiliation as a specific and often traumatic way of exercising power, with a set of consistently occurring elements and predictable consequences, including a loss of the ability to trust others. It is argued that these consequences are serious and long-lasting. The article makes a distinction between ‘shame’ as a state of mind and ‘humiliation’ as an act perpetrated against a person or group. The interplay between humiliation and shame after a humiliating act is discussed. It is argued that the patient's recovery of the capacity to resume a relatively normal life is made more likely if the therapist acknowledges the specificity of humiliation, the impossibility of reversing a humiliating act and the importance of focussing on the consequences of humiliation.

## Introduction

Hartling and Luchetta (1999) describe humiliation as ‘a relational form of human behaviour stemming from interpersonal dynamics that cannot be adequately explained by individualistic, intra-psychic theories’ (p. 260). In this article, I suggest that there is a need to see humiliation as an act which objectively takes place and which has a victim whose suffering is likely to be substantial and long-lasting. This perspective makes a distinction between humiliation and related concepts such as shame. I argue that psychotherapists risk pathologising individuals by overly concentrating on the patient's internal world and by treating humiliation and shame as though they were identical phenomena.

Approaching this subject from a multi-disciplinary perspective involving history, literature, philosophy, sociology, anthropology as well as psychology and psychoanalysis, I wish to acknowledge that psychodynamic thinking has not been central to my study of humiliation and its consequences (Leask, 2012). The readers of the journal are likely, therefore, to be experts in a field in which I am not. However, my aim is to indicate that acts of humiliation, at whatever level or in whatever circumstances they occur, consistently contain the same elements and have similar consequences, even if the extent of suffering or the ability to reduce the impact of the act of humiliation will vary, in part because of the resilience built in by successful early relationships and in part because of strategies of resistance (which themselves may owe much to such early relationships). I also suggest that recognising the specific nature of humiliation has implications for the relationship between the therapist and the patient.

It is not always easy to know from a patient's account whether an act of humiliation has or has not taken place. Often what has happened is unclear and the possibility needs to be acknowledged that the ‘victim’ may be someone continually drawn to abusive and humiliating relationships. In such situations, psychodynamic therapists would see their role not only as working with the external factors – the alleged acts of abuse and their effects – but also as seeking to understand the unconscious factors (including the influence of early experiences which might themselves have involved humiliation) that have led the patient to become repeatedly entangled in such relationships (van der Kolk, 1996, p. 183).

In this article, when referring to victims of humiliation, I have chosen to use ‘he’ because the use of ‘she’ can evoke a sense of women as victims, particularly of men; this is unhelpful in attempting to understand the nature of humiliation and its consequences. A full discussion of the gendered aspects of humiliation and their significance is outside the remit of this paper. Readers will be aware that girls and women are disproportionately represented in cases of sexual abuse and domestic violence (Herman, 2009, p. xiv). However, in many other circumstances, boys or men are the victims of humiliation.

## Humiliation: the impact of the ‘first blow’

The Austrian-born writer Jean Améry, a Jewish refugee in occupied Belgium, was arrested in 1943 by the Gestapo for distributing leaflets condemning Hitler and the war. He was immediately subjected to physical humiliation: he was brutally beaten by the police and then hung from a hook with his arms behind his back so that his joints came apart with excruciating pain as he was interrogated by the SS. The impact of this, he says many years later, remains with him and will always be something he has to live with; the act of humiliation happened and, along with the emotions and consequences flowing from it, cannot be made not to have happened. Trying to make sense of this for himself, Améry (1980/1999) says that usually when someone is injured there is also the expectation of help, which compensates for the injury. An act of humiliation, however, demonstrates the futility of such an expectation: ‘with the first blow from a policeman's fist, against which there can be no defence and which no helping hand will ward off, a part of our life ends and it can never be revived’ (p. 29). What is lost is ‘an element of trust in the world’ and the certainty that

by reason of written or unwritten social contracts the other person will spare me – more precisely stated, that he will respect my physical, and with it also my metaphysical being. The boundaries of my body are also the boundaries of my self. (p. 28)

Améry says that such an experience (which was followed by further humiliation in concentration camps) ‘blocks the view into a world in which the principle of hope rules’ and makes the victim of humiliation a ‘defenceless prisoner of fear’ (p. 40). Although Améry could be seen as displaying symptoms of ‘complex posttraumatic stress disorder (PTSD)', resulting from chronic or long-term traumatisation (Courtois & Ford, 2009; Herman, 1992/1997; van der Kolk, 1996), it is significant for a discussion of humiliation that it is the first brutal act which transforms his sense of his position in the world.

## Defining humiliation

In common usage, humiliation appears to mean much the same as embarrassment or shame or ignominy. This reflects uncertainty over what humiliation is conceptually, whether it is an act, an emotion or perhaps both. The case of Jean Améry points to power being central to humiliation, and specifically to power being used both demonstratively and unjustly. It also suggests that the likely consequences of humiliation are a sense of permanent loss and feelings of impotence, frustrated rage, despair and a ‘foul thirst for revenge’ (p. 70). After contending with these consequences for 35 years, Améry committed suicide in 1978.

An example such as this suggests that humiliation is an act that causes a change for the worse in the position of the victim and in the victim's feelings about himself and his relationship to the world. Since power is central to humiliation, the victim of an act of humiliation can be described not as *feeling* but as *being* humiliated, as the victim of an act of power. Humiliation is something actively done by one person to another, even if through institutions or directed in principle at groups. It is a demonstration of the capacity to use power unjustly with apparent impunity.

The definition I shall use here is that humiliation is a demonstrative exercise of power against one or more persons, which consistently involves a number of elements: stripping of status; rejection or exclusion; unpredictability or arbitrariness; and a personal sense of injustice matched by the lack of any remedy for the injustice suffered. Such a definition makes it easier to identify when humiliation has taken place, to understand the feelings that result from humiliation and to distinguish humiliation from shame. Humiliation leads to a strong sense that one has been wronged, while shame involves a sense that one has done wrong and diminished oneself in one's own eyes or in the eyes of others. Additionally, as Hartling and Luchetta (1999) suggest, ‘shame can serve an appropriate adaptive function by inhibiting aggression or protecting an individual from unnecessary personal exposure. In contrast, humiliation has not been identified as serving an adaptive function’ (p. 263).

## Power, rejection and exclusion

What is overtly if not always consciously demonstrated in an act of humiliation is the inequality between the person with the power and the person without it. The Savile case – the abuse of children by a television ‘personality’ who had the power to carry out the abuse and who considered that his own power made him untouchable – is an obvious example.

This exercise of power consistently involves rejection or exclusion, from a family, from a society (for refugees, for instance), from a world where trust had a meaning. Although it is not always immediately apparent, at least to the outsider, the rejection or exclusion involved in humiliation is absolute, whatever comes after. The act of humiliation has happened and, along with the emotions and consequences flowing from it, cannot be made not to have happened. Améry says he is left with ‘resentments’ and that resentment ‘nails every one of us onto the cross of his ruined past. Absurdly, it demands that the irreversible be turned around, that the event be undone. Resentment blocks the exit to the genuine human dimension, the future’ (p. 68). Améry identifies the loneliness of the victim as being at the heart of this problem; the victim is alone, excluded from the past he wants to return to, the society he believed he belonged to, and the future he expected would be his. It can be argued that a single act of humiliation in an exceptional context, such as an attack in a foreign country, does not provoke a continuing sense of exclusion or rejection. The victim returns home to somewhere safe, to trusting relationships, and is no longer threatened by the humiliator. However, the sense that the world is not as he previously understood it to be and that he is vulnerable to unexpected, arbitrary acts by someone with more power, will persist (Reiker & Carmen, 1986, p. 367). Recovery is certainly possible and ordinary life can be resumed, but as Herman says, talking of trauma: ‘recovery is never complete. The impact of a traumatic event continues to reverberate throughout the survivor's lifecycle’ (Herman, 1992/1997, p. 211).

The same pattern can be seen in cases of child sexual abuse by the Catholic Church. Clemenger's memoir (2012) is centred on his physical and sexual humiliation by the Christian Brothers at an ‘Industrial School’ in Ireland from 1959 to 1967. During this period, he felt excluded within the school from any kind of ordinary life, and outside the school from what he saw as ordinary families with happy children. To save himself from the worst of the physical violence, he had to accept the protection of two parental figures who seemed genuinely fond of him but who were his sexual abusers. He understood how destructive this was: ‘I was an outsider, driven by hate and isolation’ (p. 100). His story as he grew up involved a suicide attempt, further rejection and discrimination after leaving the school, the refusal of most authority figures to believe his account of his abuse, time in prison, profound suspicion of the motives of people who were kind to him, and eventual rescue by the woman who became his wife. Like Améry, he tried to put the humiliation behind him, adopting a thought-out approach to managing its consequences. He avoided introspection, refused to believe he was responsible for his abuse, accepted there would still be ‘bad days’, pursued his education, invested in his emotional life with his wife, and remained determined to be optimistic and ‘less occupied with “if only”’ (pp. 346–347). However, when the child abuse scandal became public in Ireland in the 1990s, the memories he had been hiding from for decades ‘broke free of their chains and began to torture me night and day’, making it impossible to sleep and causing him to become dependent on anti-depressants (pp. 356–357).

For Michael Clemenger, even rediscovering the family he thought he should be part of was an unsuccessful attempt to be included again, to overcome the original rejection that caused him to be raised in church institutions. For children abused within the family, there can similarly be no return to a past where they want to be, belonging at the heart of the family, able to love and trust unconditionally (Goodyear-Brown, 2012, p. 18).

As Améry notes, humiliation compromises the future. Wayne Koestenbaum (2011), in a deliberately fragmented essay, provides in the last pages one long example of humiliation and dedicates his book to its victim (pp. 203–206). In a queue at an airport, Koestenbaum recounts, a man kicks his daughter in the buttocks. She is ‘pubescent’ and probably thirteen years old. She is enraged and bursts into tears, but also looks remorsefully at her father, whose expression remains cold. Koestenbaum, the observer, wants to console the daughter, but feels, against his better judgment,

a rueful, despairing pity for the father, who'd destroyed any possible future chance of happiness for himself and for his daughter, and who did not seem aware that he had poisoned the future, like spilling oil into an ocean and never being able to clean it up.

The father, with his ‘violent yet enigmatic act’ of humiliation, has destroyed the possibility of a future where there could be safety and mutual trust.

## Unpredictability, arbitrariness and injustice

Humiliation almost always happens unexpectedly, even if the victim has been living in fear of it. It involves a breach of law, norms or values that both the humiliator and the victim believed were binding. The parent sexually abusing a child is doing something the child, even in his confusion, senses is in conflict with all that he has been brought up to believe about what is right and to be expected from a parent. In 1935, Jewish German citizens found their lives transformed from one day to the next by the Nazis’ Nuremberg Laws, which stripped them of their status as Germans. Michael Clemenger was abused by people who hypocritically railed against the very acts they were committing.

Unpredictability reinforces the power of the humiliator and inculcates a fear of humiliation which is powerful in itself. This can be reinforced when people have witnessed or heard about acts of humiliation (Hartling & Luchetta, 1999, p. 262). In the examples quoted above, all the victims were vulnerable to arbitrary or unpredictable acts by those in power. Since those in power also controlled the justice system and denied access to it to those they humiliated, a sense of helplessness in the face of injustice was central to the victims’ response to humiliation.

## Humiliation and resistance

Can humiliation be refused or rejected by the intended victim? Because of the power relations involved, this appears unlikely. A partial exception arises when people are engaged in resistance activities which demonstrate that they do not accept or share the norms and values of those in power. Communists in Nazi Germany believed they might be killed for their attempts to resist the Nazis, but not that they could be humiliated. Jehovah's Witnesses imprisoned in the German Democratic Republic (GDR) displayed an extraordinary capacity for resistance while waiting for a better life after this one (Kabelitz, 1939–1956, p. 292). In such cases, resisters see their punishment and exclusion as predictable consequences of the power struggle they are involved in. They see themselves as temporarily defeated, not as victims of humiliation. Faced with such resistance, those in power frequently respond with acts of torture or other ‘cruel and unusual punishment', in order to demonstrate that they can humiliate even those who deny that this is possible.

Herman (2009) lists the actions that perpetrators use to humiliate a victim and suggests that the consequences of such actions can include PTSD or complex PTSD (p. xiv). Where resistance as a way of staving off humiliation is successful, the struggle to resist may in itself be traumatic. However, such resistance might also reduce the incidence of PTSD. In their study of former GDR political prisoners, Ehlers, Maercker, and Boos (2000) highlight ‘mental defeat’ but also alienation and feelings of permanent change as likely indicators of PTSD among prisoners after their release. Mental defeat, contrasted with ‘the perception of oneself as an autonomous human being’ (p. 45), was common and is a logical consequence of humiliation. It showed up in the study as the strongest predictor of the severity of subsequent PTSD symptoms. In other words, mental defeat is more likely even than ‘perceived threat to life’ to lead to severe PTSD symptoms. The authors note that the results are in line with their hypothesis (which is also mine) that ‘perceived threat to one's psychological autonomy is an important aspect of the psychological severity of trauma that is intentionally inflicted by other people’ (p. 51). Significantly, the study suggests that resistance based on political commitment and understanding leads to a better long-term outcome in relation to the impact of potentially traumatic events and the likelihood of depression. This provides little solace, however, to the vulnerable individual victim of humiliation, particularly a child in a family or in other settings where those in authority misuse their power. Here, any desire to resist is compromised by the huge imbalance of power, physically, emotionally and socially, and the ambivalent attitude of the child towards the parental figure (Philpot, 2009, pp. 105–106).

## Consequences of humiliation

Humiliation, except in trivial cases that we tend to shrug off, can have a life-changing effect on the victim, as the examples above suggest. Another well-known example arises from the brutal humiliation of Rodney King, a black man, by four white police officers in Los Angeles in 1991. The initial acquittal of the officers, widely interpreted as an indication that the state and the society condoned the humiliation, led directly to riots in which over 50 people died. A *Guardian* interview with King in May 2012 portrays him as asserting his happiness at being alive and being able to talk about his story, and as suddenly lapsing into ‘his memory's darkest recess’ as he relives the attack (Carroll, 2012). He shows signs of ‘decades of alcohol abuse and numerous car accidents’ and is ‘a forlorn figure seemingly trapped by his past, his name and his addiction to alcohol, all, in his mind, inextricably bound'. The interviewer notes King's consuming anger and the ‘self-destructive vortex that cost him family, health and savings’ in the years after the humiliation. There is every indication that King's problematic childhood had left him poorly equipped to deal with the attack on him and its aftermath. King's own view was that time heals and that he had finally found peace. Six weeks later, King drowned, apparently accidentally, in his swimming pool, while heavily intoxicated by alcohol and a variety of drugs.

A sense of invasion of the sort King experienced, of personal boundaries illegitimately crossed and of the self being diminished as a result, is central to the personally destructive power of humiliation. Ripstein (1997) says that the response of the state through the legal system is important, something that is relevant to the King case as well as to the examples of child abuse. Punishment, Ripstein says, ‘serves to avoid society acquiescing in that humiliation. […] To fail to punish a crime is to turn a private humiliation into a public one’ (p. 103). Of course, that is the explicit intention when the state itself deliberately engages in humiliation, as in Nazi Germany.

## The victim's responses

Any act of humiliation may be experienced as traumatic but, as is reflected in the psychoanalytic discussion of trauma, different influences and background experiences, particularly early relationships and the ways in which these have been internalised, influence how individuals react when they become the victims of traumatic humiliation (Baron-Cohen, 2011, pp. 47–48; Bentovim, Cox, Bingley & Pizzey, 2009, p. 12; Gaskill & Perry, 2012, pp. 29–30; Goodyear-Brown, 2012, pp. 14, 18–19; Krystal, 1988; Philpot, 2009, pp. 11–14; van der Kolk, 1996, pp. 185, 202).

Personal accounts of humiliation suggest that the victim tends to pass through different sets of responses, from a sense of bewildered helplessness to rage and from there to revolt, resistance or submission, which may also involve despair and self-destruction. The first stage frequently involves surprise and shock at what has happened, dismay and disorientation because of the rejection or exclusion involved, grief at the loss sustained and bewilderment at the injustice suffered. How is a child to make sense of an abusive parent, a woman to come to terms with the realisation that a loving husband can be mercilessly violent towards her?

The next stage is likely to involve rage and a desire to lash out and seek revenge. For the victim of humiliation, the sense of injustice is a primary cause of rage. When humiliation affects a whole society or a large group in it, those Lindner refers to as ‘humiliation entrepreneurs’ (2006, p. xv) use this rage at injustice and the sense of impotence that flows from it as a way of building support for violent, retaliatory action. (Hitler's use of the perceived humiliation of Germans resulting from the Versailles Treaty is an obvious example.) Anger, hate and violence are psychologically damaging to the victims of humiliation, and a cycle of humiliation and retaliation may be set up, leading to yet further suffering and destruction. This applies not just at the level of national and international politics; as is often noted, it is not uncommon for victims of child abuse to become abusers themselves in later life (Bentovim et al., 2009, p. 68; Goodyear-Brown, 2012, p. 12).

The anger resulting from humiliation might also be matched by a realistic sense of powerlessness. Responses to this include strategies of avoidance: looking away from reality; self-deception over what has happened; and refusing to face up to the new, reduced circumstances (Philpot, 2009, p. 14). The victim may become indifferent to the fate of others around him, or actively cruel, since this restores some sense of power to him.

Despite the differences between them, there is often an interplay between humiliation, shame and guilt, which is important to note when considering the consequences of humiliation. The avoiding action taken by people fearing humiliation can lead to them doing things they accept, internally, are wrong, for which they feel shame: joining in with the humiliation of others, for instance. Resorting to a sense of shame is also a way of seeking to control what is uncontrollable by admitting or claiming one's part in it: the victim blames himself for doing wrong, not the person who has wronged him. Similarly, feelings of guilt imply an acceptance of an external authority with agreed rules, such as a parental figure; since the rules have been broken, the victim accepts that the authority is entitled to punish him. Feeling guilt (like feeling shame) in response to humiliation is a way of trying to make sense of the inexplicable, of trying to impose a pattern on what otherwise appears as random, arbitrary behaviour. This is particularly common in childhood. It is safer, psychologically, for a child to see himself as a bad child, rather than as a child with bad parents. In doing so, he is able to cling to a sense of basic fairness and to avoid admitting the injustice of the humiliating acts. Blaming himself at least provides an explanation for what has happened (Philpot, 2009, p. 13). As it also excuses the humiliator, it is in the interest of the humiliator to develop a sense of guilt or shame or both in the victim (Smith, 2008, p. 373).

The sense of powerlessness among victims of humiliation can lead to paranoia, despair or depression. For Koestenbaum, ‘humiliation's wounds are always intimate, pointed punctures'. One response to this involves closing off from the world physically and psychologically while metaphorically creating a hard skin or shell to control what is allowed in or out (Bick, 1968; Turp, 2007).

When none of the coping strategies proves effective and the reality of the victim's position overwhelms him, he may reach the stage of personal fragmentation and disintegration, with severe difficulties in day-to-day functioning at either an individual or a social level. This was the position reached by Michael Clemenger and Rodney King even after each of them thought he had successfully let go of the effects of his humiliation.

## Professional responses

Inevitably, in the course of their work, therapists will find themselves face to face with the personal consequences that arise from living with the contradictory feelings, experiences and defences resulting from humiliation. The therapist's reading of the situation is likely to have profound implications for his or her practice. Just as professional intervention in cases of child abuse involves ensuring the child is safely out of reach of the abuser – the humiliator – so the therapist will, at the very least, seek to provide a safe space in the consulting room for the humiliated adult patient. However, he or she may also be faced with the knowledge that the patient is still under the control of the humiliator and that the humiliation may be continuing. A number of examples are given below of how therapists see themselves contending with the consequences of the patient's humiliation.

Lewis (1987) uses the terms ‘humiliated fury’ and ‘shame-rage’ as synonyms, in a way that is suggestive of the consequences of humiliation more than of shame. However, she does not recognise the specific consequences (including shame) that result from humiliation. Her view that shame is ‘seen as a means by which people try to preserve their loving relationships to others’ (p. 2), can apply when the victim of humiliation seeks to shift the blame from the humiliator to himself but it denies the underlying power relations involved in humiliation.

Moses-Hrushovski (1994), while acknowledging that her humiliated patients have suffered terribly from brutality or sexual abuse, nevertheless classifies their defences as ‘pathological’ (p. xix). Frequently treating the terms shame and humiliation as synonymous, she suggests that the blaming processes used by her patients ‘are efforts to shift the direction of shame and guilt away from themselves in order to avoid painful experiences’ (p. 6). There is a danger here of blaming the victim and therefore humiliating him again – at which point the consulting room ceases to be a safe space – rather than helping him to mourn the losses engendered by the experience of being humiliated. As Jean Améry said about the suggestion that his preoccupation with anti-semitism and the Holocaust meant he was mentally ill or suffering from hysteria: ‘I know that what oppresses me is no neurosis, but rather precisely reflected reality. Those were no hysteric hallucinations when I heard the Germans call for the Jews to “die like a dog!”’(p. 96).

Gilbert (1998) (a cognitive behavioural therapist) notes overlaps and differences between shame and humiliation, including powerlessness and a sense of injustice in humiliation, and suggests that ‘in humiliation-based problems there is a focus on the harm done by others’ (p. 259). Discussing possible therapeutic interventions, he says that some patients may fear their destructive actions or loss of control and that for them it can be useful ‘to explore ways of gaining emotional control and make it clear that in working with humiliation this is not an encouragement to act out these feelings but to heal them’ (p. 263). Many of Gilbert's proposed interventions seek to deal not so much with the humiliation as with the rage and hatred arising from it, as if this is ultimately all that can be done. Gilbert implicitly accepts this when talking, apparently approvingly, of a patient considering leaving a job where he was being humiliated (p. 264). For the patient, such a move represents an admission of powerlessness and a clear victory for the humiliator; there is no justice involved, only the possibility of starting again elsewhere and trying to manage the remaining feelings of rage and hatred or, in this case, depression, arising from the humiliation.

Ehlers et al. whose study highlights the long-term effects of humiliating acts and the possibility of permanent change for the victim, offer somewhat unconvincing suggestions for treatment. Among these, they propose that patients who have ‘an overall feeling of alienation or permanent change’ could benefit from ‘interventions that encourage them to re-establish contact with friends and family and to take up activities again that they used to enjoy before the trauma’ (p. 54). For someone such as Jean Améry, Michael Clemenger or Rodney King, these can hardly be seen as effective routes to recovery.

The psychoanalyst Phil Mollon, in a study of shame and related emotions (2002), writes that ‘the cure for states of shame and humiliation is empathy’ (p. 20), but does not specifically look at the meaning and significance of humiliation. He describes the case of ‘Natalie’ whose mother ‘appeared to have been highly invasive and controlling, insisting that her daughter have no secrets from her’ (p. 6). Against her own will and better judgement, Natalie ‘tended to structure her life around lies and deception’ (p. 8) as a defence against a ‘violation of her core self’. Mollon says that such violation ‘can be experienced as a rape of the mind – indeed of the soul – and as damaging, potentially, as a physical rape. The emotional response to violation is shame’ (p. 7). Another way of expressing this would be to say that Natalie grew up with a constant and justified fear of humiliation, since all the elements of humiliation are present here: the invasion or violation is a demonstrative exercise of power that conflicts with the apparently accepted norms of behaviour between a mother and her daughter and involves rejection, the arbitrary application of rules Natalie cannot agree with and injustice without remedy.

Natalie's lies and deception, therefore, are the means she uses to defend herself against humiliation by her mother. Mollon recognises that this gives her a sense of freedom and agency, since it indicates she retains a private core self. Natalie's problems involve an unrealistic transference to others whom she sees as equivalent to her mother. The indiscriminate use of lies that results from this seriously damages her other relationships. Mollon says that part of the therapeutic work ‘was for Natalie to discover that honesty did not have to mean violation of her core self – and to realise that whilst she certainly had the capacity to lie and conceal successfully, she could *choose* to be truthful’ (p. 9). It is here that it would be helpful for the therapist to acknowledge also that Natalie had been repeatedly humiliated and was not responsible for her mother's invasive behaviour. This could then lead Natalie to discover something different: choosing to be truthful *to everyone but her mother* need not necessarily open her up to attacks from others and could instead be a way of responding to and overcoming the feelings of shame that have arisen when she has avoided telling the truth in the past. In order to hold her humiliating mother at arm's length, it might be necessary to continue lying to her and for Natalie to recognise that she need not feel shame for this defence against her mother's continuing attempt to violate her core self.

Hartling and Luchetta suggest that when it is clear how much humiliation or the fear of humiliation is contributing to a patient's psychological problems, treatment could focus on ‘untangling and resolving the debilitating consequences of the individual's actual or perceived experience; restoring the individual's sense of self to a more optimal level of self-respect and self-worth'. The therapist should help to strengthen ‘the individual's resistance or resilience in the face of possible, frequently inevitable, future humiliations’ and to empower him to ‘challenge and change social and environmental factors that are likely to support or promote humiliating social practices’ (p. 273).

## Conclusion

I have argued here a number of theoretical points about the nature of humiliation. Firstly, I suggest that humiliation is a specific way of exercising power with a specific set of responses and consequences that are often catastrophic and life-changing. Secondly, I argue that humiliation is an act of power, demonstratively and unjustly used with apparent impunity, and that humiliation is not an emotion in itself and therefore not to be confused with shame, but that it leads to a predictable set of emotions which may at times include shame but in which rage and the desire for revenge, combined with a sense of impotence, tend to dominate. In line with many other theorists, I also argue that acts of humiliation cannot be made not to have happened and that their emotional impact is likely to persist over the long term. At the same time, I acknowledge that the both the degree of suffering arising from an act of humiliation and the capacity to move on from such an act and to rebuild one's life varies from person to person, partly at least in accordance with the inner strength and resilience that arise from successful early relationships or from strategies of resistance.

All of these theoretical points have implications for therapists in their work. They imply that it is important for therapists to have a dual focus. Firstly, as van der Kolk, McFarlane, and Weisaeth (1996) state in support of the psychodynamic approach to treating the victims of severe trauma, therapists need to ‘focus on understanding the subjective meaning of the traumatic event and the process of (and barriers to) the integration of the experience with pre-existing attitudes, beliefs and psychological constructs’ (p. xvii). Secondly, they need to accept that a patient's stories might include fantasies and delusions but might also contain accounts of real, terrible suffering at the hands of someone else, and of injustice that cannot be remedied.

Where the humiliating acts are in the past, the patient has a chance to mourn what he has lost rather than engage in a futile search for restitution or what in German is called *Wiedergutmachung*: making good again, restoring things to the way they once were. He can then be helped to shed his sense of being a victim and move forward towards re-establishing trust in others and a sense of autonomy, but without denying the continuing impact of what has been done to him and the knowledge that it can never be undone.

Where, however, the humiliating acts are continuing – where, that is, the extreme imbalance of power has not been altered – the patient may have a realistic sense that he continues to be a victim whose capacity to act autonomously is still heavily compromised. The therapist is faced with the question of how to respond to this unresolved situation, where what Gilligan (2000) calls the outside forces over which the patient has no control contribute to ‘the range of problems that are not amenable to being solved by self-knowledge, though some of them might be solvable by other means, such as medical research or political action’ (p. 21). Here, it may be helpful for the patient to identify the possible options – which may themselves have painful, disruptive consequences – for bringing the humiliating relationship to an end or escaping from it, or to reach an understanding of why he persists with or even takes refuge in relationships that are humiliating.

In either case, it is important, as van der Kolk (1996) says, for therapists to identify and accept the ‘essential truth’ of their patients’ experiences of past trauma – in this case, humiliation – so that they do not ‘aggravate feelings of rage and helplessness by invalidating the realities of their patients’ lives’ (p. 183). Herman (1992/1997) is more insistent, asking the therapist to ‘bear witness to a crime’ and to adopt a position of solidarity with the victim, without necessarily absolving the victim of all blame, a position which ‘involves an understanding of the fundamental injustice of the traumatic experience and the need for a resolution that restores some sense of justice’ (p. 135).

Recognising the specific nature of humiliation, the therapist can provide the necessary place of safety in which the patient can start to think about and articulate what it means to be a victim of humiliation. In this place of safety, the patient needs to know or sense that the therapist will not deny the reality of his experiences, will not seek to treat him as someone for whom shame is or should be the central emotion arising from these experiences and will not seek to impose on him a sense that everything that has been done to him can be put behind him. The therapist will recognise humiliation for what it is: an exercise of power that is demeaning, arbitrary, excluding and unjust and which can never be made not to have happened.

## References

[R1] Améry J., Rosenfeld S., Rosenfeld S. (1999). At the Mind's Limit. Contemplations by a Survivor on Auschwitz and its Realities.

[R2] Baron-Cohen S. (2011). Zero degrees of empathy. A new theory of human cruelty.

[R3] Bentovim A., Cox A., Bingley M. L., Pizzey S. (2009). Safeguarding children living with trauma and family violence. evidence-based assessment, analysis and planning interventions.

[R4] Bick E. (1968). The experience of the skin in early object-relations. International Journal of Psycho-Analysis.

[R5] Courtois C. A., Ford J. D. (2009). Treating complex traumatic stress disorders. An evidence-based guide.

[R6] Carroll R. (2012, May 2). ‘I had to forgive’: interview with Rodney King. The Guardian..

[R7] Clemenger M. (2012). Everybody knew. A boy, two brothers. A stolen childhood.

[R8] Ehlers A., Maercker A., Boos A. (2000). Posttraumatic stress disorder following political imprisonment: The role of mental defeat, alienation, and perceived permanent change. Journal of Abnormal Psychology.

[R9] Gaskill R. L., Perry B. D., Goodyear-Brown P. (2012). Child sexual abuse, traumatic experiences, and their impact on the developing brain. Handbook of child sexual abuse: Identification, assessment, and treatment.

[R10] Gilbert P., Tarrier N., Wells A., Haddock G. (1998). Shame and humiliation in the treatment of complex cases. Treating complex cases: The cognitive behavioural therapy approach.

[R11] Goodyear-Brown P. (2012). Handbook of child sexual abuse. Identification, assessment, and treatment.

[R12] Gilligan J. (2000). Violence: Reflections on our deadliest epidemic.

[R13] Hartling L. M., Luchetta T. (1999). Humiliation: Assessing the impact of derision, degradation, and debasement. Journal of Primary Prevention.

[R14] Herman J. L. (1997). Trauma and recovery..

[R15] Herman J. L., Courtois C. A., Ford J. D. (2009). Foreword. Treating complex traumatic stress disorders. An evidence-based guide.

[R16] Kabelitz F. (1939–1956). Lebenserinnerungen..

[R17] Lewis H. B. (1987). The role of shame in symptom formation.

[R18] Koestenbaum W. (2011). Humiliation.

[R19] Krystal H. (1988). Integration and self-healing: Affect, trauma, alexithymia.

[R20] Leask P. (2012). Power, the party and the people: The significance of humiliation in representations of the German Democratic Republic (PhD thesis).

[R21] Lindner E. (2006). Making enemies. Humiliation and international conflict.

[R22] Mollon P. (2002). Shame and jealousy: The hidden turmoils.

[R23] Moses-Hrushovski R. (1994). Deployment: Hiding behind power struggles as a character defense.

[R24] Philpot T. (2009). Understanding child abuse. The partners of child sex offenders tell their stories.

[R25] Reiker P. P., Carmen E. H. (1986). The victim-to-patient process: The disconfirmation and transformation of abuse. American Journal of Orthopsychiatry.

[R26] Ripstein A. (1997). Responses to humiliation. Social Research.

[R27] Smith D. (2008). Globalization, degradation and the dynamics of humiliation. Current Sociology.

[R28] Trumbull D. W. (2008). Humiliation: The trauma of disrespect. Journal of the American Academy of Psychoanalysis and Dynamic Psychiatry.

[R29] Turp M. (2007). Self-harm by omission: A question of skin containment?. Psychodynamic Practice.

[R30] van der Kolk B. A., van der Kolk B. A., McFarlane A. C., Weisaeth L. (1996). The complexity of adaptation to trauma. Traumatic stress: The effects of overwhelming experience on mind, body, and society.

[R31] van der Kolk B. A., McFarlane A. C., Weisaeth L. (1996). Traumatic stress. The effects of overwhelming experience on mind, body, and society.

